# Frictional Properties of Soaps with the Addition of Ground Hazelnut Shells

**DOI:** 10.3390/ma17122966

**Published:** 2024-06-17

**Authors:** Jacek Mazur, Monika Wójcik, Renata Różyło, Paweł Sobczak, Marian Panasiewicz, Sławomir Obidziński

**Affiliations:** 1 Department of Food Engineering and Machines, University of Life Sciences in Lublin, 28 Głeboka St., 20-612 Lublin, Poland; monika.wojcik@up.lublin.pl (M.W.); renata.rozylo@up.lublin.pl (R.R.); marian.panasiewicz@up.lublin.pl (M.P.); 2 Department of Agri-Food Engineering and Environmental Management, Bialystok University of Technology, 45E Wiejska St., 15-351 Białystok, Poland; s.obidzinski@pb.edu.pl

**Keywords:** hazelnut shells, biological materials, soap, cosmetics, sustainability development

## Abstract

The search for new technologies and related new biological materials for use in the cosmetics industry requires many studies and analyses of not only chemical but also physical properties. This study attempts to assess the properties of soap produced with the addition of crushed hazelnut shells. This additive is intended to improve the friction properties of the soap, which in turn enhances the quality of removing impurities from the skin. Friction tests for wet and dry skin were performed on an appropriately designed measuring station using the Texture Analyser XT plus device. The obtained results indicate an increase in dynamic friction value compared to the control sample. This work proposes an unconventional use of ground hazelnut shells as one of the additives in soap production to improve its quality.

## 1. Introduction

The traditional bar of soap is becoming more and more popular not only on the personal hygiene products market, but also on the ecological products market [1]. This is mainly due to the commercial use of soap and additional functions, such as a skin-softening deodorant, moisturizer, and bacteria-reducing agent [2,3]. The possibility of using various waste materials, such as almond shells, orange peels [4], or used cooking oil to make everyday soaps can increasingly be recognised [5].

Population growth, as well as changes in lifestyle and dietary patterns, are the main reasons for increased waste production, resulting in a range of environmental and public health side effects [6]. Post-production and food waste can be generated throughout the entire food supply chain, i.e., starting with food production and ending with its serving. The accumulation of increasing amounts of waste is becoming a global problem due to the continued growth of food and industrial production. The rapid increase in waste production poses serious risks to society, causing environmental pollution, health risks to the population and overloaded landfills, to name a few. Post-production and food waste has become a priority on both the global and national political agenda. This is borne out by the UN Sustainable Development Goals (Goal 12—responsible consumption and production) and the European Commission’s action plans for a circular economy.

Hazelnut shells, which are by-products of harvesting edible parts for food, can be used as an unconventional additive to soaps. Hazelnut shells make up more than half of the total weight of the fruit, and their composition consists mainly of lignins (equal to about 50%) [7,8]. Shells as a by-product are so far commonly used as an energy fuel (combustion) [9] and less commonly used as a raw material for the production of furfural in the dye industry [10] or as a ground bedding for the cultivation of certain crops [11]. Other studies point to the use of hazelnut shells in combination with orange peel and rice husks as ingredients that enrich the soil with minerals for rice cultivation and have the potential to reduce the amount of heavy metals in soil to levels below acceptable limits [12]. In addition, hazelnut shells have been proposed as an additive in combination with melamine-urea formaldehyde or polyurethane for making particleboard [13]. There have also been attempts to use hazelnut shell powders to produce biodegradable Mater-Bi composites [14] or polystyrene composites [15].

No research has been conducted on using powdered hazelnut shells for soap making. Due to the granular structure of these powders, they have been proposed as soap additives that have an exfoliating effect. In general, a scrub is a substance added during soap manufacture to cleanse dead skin and impurities on the skin, making the skin brighter [16], and by introducing hazelnut shells into soap, a scrubbing effect can be achieved, thus helping to cleanse the skin of impurities. In previous research, the friction qualities of soaps were not a common topic. Only our prior study presented a method for measuring friction qualities of soaps without additives [16,17,18] and soaps including apple and carrot pomace [19]. Other soap tests were conducted on soap samples that included Citrullus lanatus, Citrus lemon, Citrus maxima, Carica papaya, Ananas comosus, and Punica sekretum fruit peels [20]. The antibacterial qualities and pH of the produced soaps were tested, but no friction properties were measured. Other studies on rheological (viscoelastic behaviour) and textural (firmness) properties have shown that natural soaps obtained from chestnut husk ashes can be interesting products for the soft soap market and provide additional economic value due to the reuse of by-products [21]. Other studies investigated transparent soap composition containing sappan wood extract, including organoleptic testing, pH, humidity, foam stability, wetting power, and formulation hardness. However, there was no assessment of soap friction properties [22]. Considering the above, the frictional properties of soap with ground hazelnut shells were tested.

## 2. Materials and Methods

### 2.1. Research Material

The test material used in the measurements consisted of soaps made from a transparent glycerin base (Forbury, Riga, Latvia), to which a 5% addition of crushed hazelnut shells was proposed. The amount of the additive was determined as a result of preliminary tests—the additive in this amount provided a texture and consistency similar to that obtained in soaps without them. The hazelnut shells used in the study came from crops from the Lublin region (Poland). The shells were ground in an impact crusher and fractionated on screens into three particle sizes: (a) smaller than 0.5 mm; (b) 0.5–1 mm; (c) 1–2 mm. The soap production technology consisted of dissolving the soap base in a water bath at a temperature of about 67 °C. The water temperature was controlled with a thermometer so that it did not exceed 70 °C, as the base heated above this temperature loses its properties. Subsequently, the melted soap base, with a weight of 5 g (±0.5 g), was poured into a rectangular mould with holes 26 mm (±0.1 mm) in diameter and 12 mm (±1 mm) in height, to which crushed hazelnut shells were previously added in the amount of 5% (0.25 g). The mixture was then stirred quickly to distribute the shell powder evenly. The soaps were left to solidify at room temperature for 50 min. The soaps were made in 10 replicates for each of the fractions analysed.

### 2.2. Friction

First, frictional resistance measurements were carried out for a dry synthetic leather and then again when the surface was wetted with distilled water (1 cm^3^ of water spread on the analysed surface 10 s before the test) at a relative humidity equal to 40% ± 5%. Additionally, to simulate contact with heavily “worked out” human skin, a 320-grit sandpaper surface was used at a relative humidity of 40% ± 5%. The load applied on the sample during the tests on each of the analysed surfaces was variable and amounted to 100, 200, and 500 g, making it possible to obtain a pressure of 1.94, 3.79, and 9.33 kPa, respectively.

**Figure 1:**
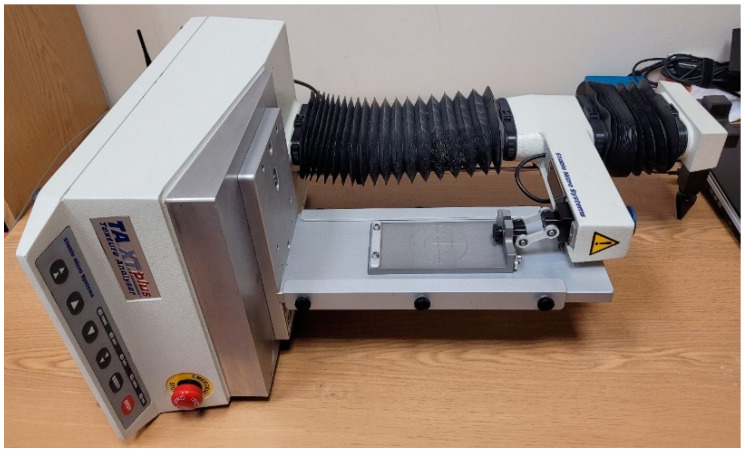
Horizontal friction measurement attachment for TA.XT plus texture analyser.

Measurements were performed on a TA.XT plus Texture analyser, made by Stable Micro Systems (Godalming, UK), using a procedure developed by the company to measure friction, called Measuring bi-directional friction properties of materials using the Horizontal Friction System (Figure 1) [23]. The measurement method was developed in accordance with the modified ASTM Standard Method D1894 [24]. The measurements were carried out on a modified friction bench with an original attachment submitted to the patent office [17,18].

Measurements of initial friction (stiction), dynamic friction (friction), and frictional work during unidirectional dynamic friction were performed for a linear distance.

During the movement of the measuring head (and the combined set with the sample) relative to the analysed surface, the forces occurring in the course of the test were recorded by the measuring head. Stiction is read as the maximum force recorded when the test set moves along the surface for the first 5 mm of travel during the test. Friction is read as the maximum force recorded when the test set moves along the surface after the initial 5 mm of movement during the test. The work of dynamic friction was defined as the area under the friction force/distance graph (the displacement of the measuring head from the sample (after the initial 5 mm of movement during the test). Linear distance is calculated as the length of an imaginary line connecting all points on the force/distance graph (after the initial 5 mm of movement during the test).

The static coefficient of friction Us was determined from the following relationship:

$$Us=\frac{stiction}{pressure\;of\;the\;sample}$$

The Us coefficient is calculated as the ratio of the maximum force recorded during the movement of the measuring set with the sample on the surface for the first 5 mm of movement during the test and the pressure force (gravity) resulting from the mass of the measuring instrument, the mass of the weight applied on the soap sample, and the mass of the soap sample itself.

The dynamic coefficient of friction Uf was determined from the following relationship:

$$Uf=\frac{friction}{pressure\;of\;the\;sample}$$

The Uf coefficient is calculated as the ratio of the maximum force recorded during the movement of the measuring set with the sample on the surface after the initial 5 mm of displacement during the test and the pressure force (gravity) resulting from the mass of the measuring instrument, the mass of the weight applied on the soap sample, and the mass of the soap sample itself.

The test speed of the sample during the friction test in both directions was 2.5 mm·s^−1^, the travel distance of the measuring platform was 100 mm, and the initial displacement before the start of dynamic friction measurements was 1 mm [18].

### 2.3. Statistical Analysis

The obtained results were subject to statistical analysis. The normality of the distribution was determined using the Shapiro–Wilk test. Basic statistics were calculated, and ANOVA analysis of variance was performed for the factors. The Tukey test was used to determine the significance of differences, assuming a significance level of *p* = 0.05. Trials were carried out in 5 repetitions.

## 3. Results

The results of the friction parameters of soap with the addition of crushed hazelnut shells obtained on various surfaces, including leather, water-moistened leather, and sandpaper, are shown in the following figures (Figure 2, Figure 3, Figure 4, Figure 5, Figure 6, Figure 7, Figure 8, Figure 9, Figure 10, Figure 11, Figure 12, Figure 13, Figure 14, Figure 15, Figure 16, Figure 17, Figure 18 and Figure 19) and tables (Table 1, Table 2, Table 3, Table 4, Table 5 and Table 6).

**Figure 2:**
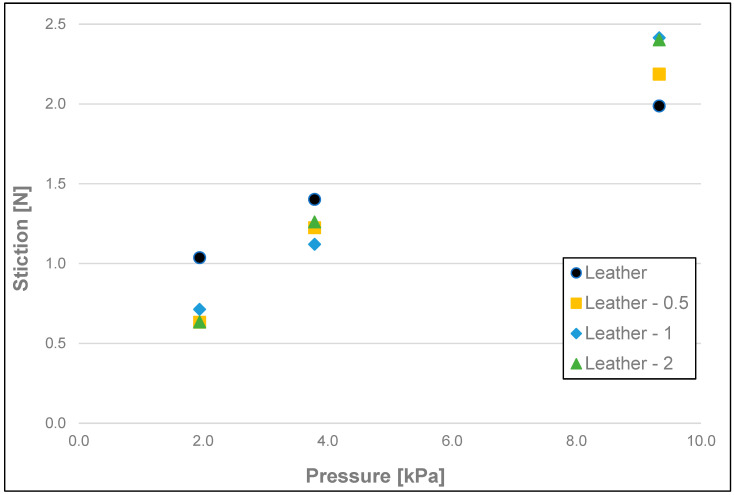
Initial friction (stiction) of analysed samples of hazelnut soap on dry synthetic leather.

**Figure 3:**
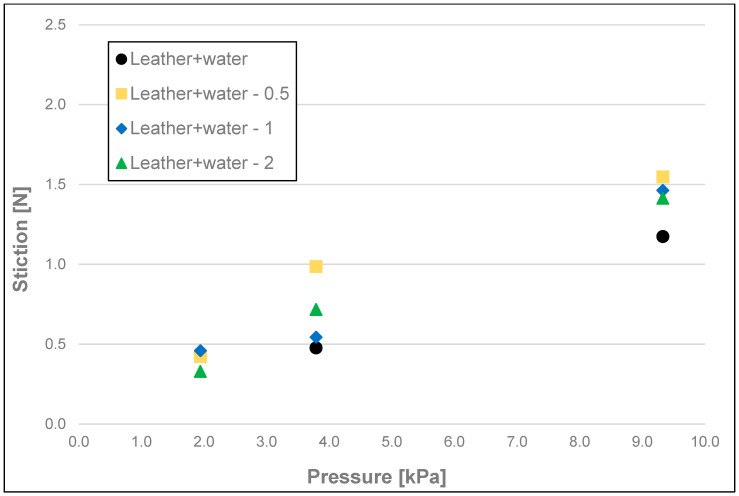
Initial friction (stiction) of analysed samples of hazelnut soap on moistened synthetic leather.

**Figure 4:**
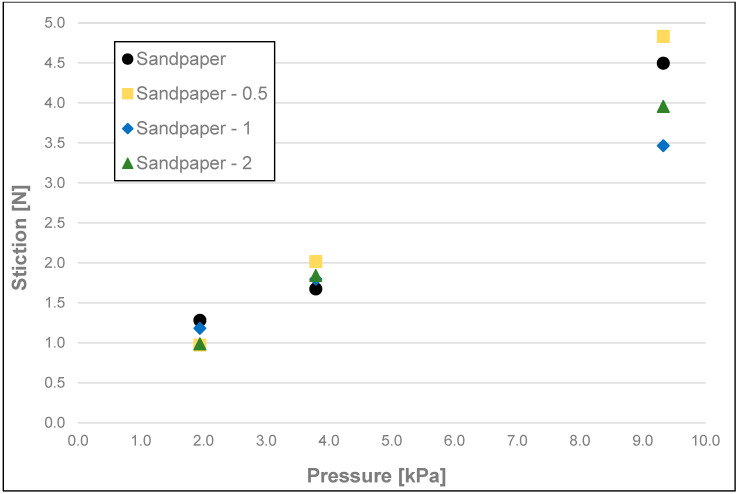
Initial friction (stiction) of analysed samples of hazelnut soap on sandpaper.

The obtained measurements of initial friction (stiction) (Figure 2, Figure 3 and Figure 4 and Table 1) of the soap showed significant differences between the friction values of the soap on dry and wetted leather and on sandpaper, at each pressure tested, i.e., 9.33 kPa; 3.79 kPa, and 1.94 kPa. However, with the sandpaper surface at pressures of 1.94 and 3.79 kPa, the values obtained on dry leather were not statistically significant. As the pressure increased for each of the surface variants analysed and for each fraction of ground hazelnut shells, there was an increase in static friction values. For soaps tested on dry leather, there were no significant differences between the highest and medium pressure (9.33 kPa and 3.79 kPa) and medium and low pressure (3.79 kPa and 1.94 kPa). No apparent significant effect on static friction could be attested for soaps with a 5% addition of crushed hazelnut shells. Differences for tested surfaces were significant at the highest pressure (9.33 kPa), where, for example, the average value of stiction for soap without added nutshells was 1.99 N for the dry leather surface, 1.17 N for the moistened leather surface, and 4.50 N for sandpaper.

**Figure 5:**
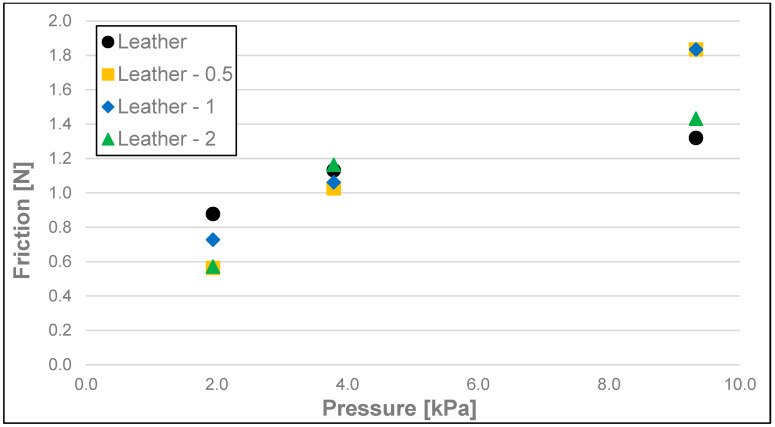
Dynamic friction of analysed soap samples on dry synthetic leather.

**Figure 6:**
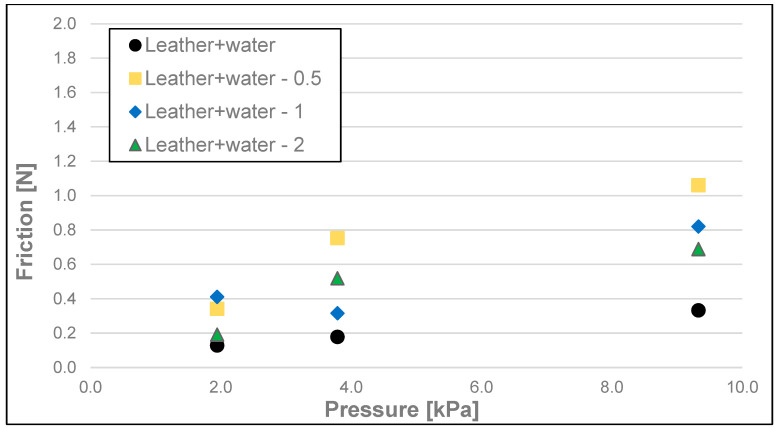
Dynamic friction of analysed soap samples on wetted synthetic leather.

**Figure 7:**
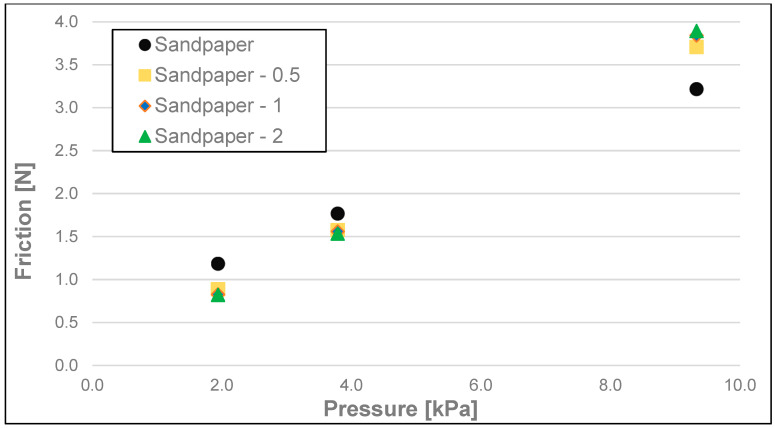
Dynamic friction of analysed soap samples on sandpaper.

Dynamic friction (friction) of the soap samples analysed as a function of load is shown in Figure 5, Figure 6 and Figure 7 and Table 2. Here, the most significant differences for each surface were observed between dynamic friction measurements at a pressure of 9.33 kPa and measurements at a pressure of 1.94 kPa. As in the case of stiction, no statistically significant differences occur when any of the nutshell fractions are added for the pressures of 1.79 and 3.79 kPa, with the only exception at the pressure of 1.94 kPa and the addition of fractions 0.5–1 and 1–2 in the case of the dry leather surface when a significant difference was recorded. In most cases, no statistically significant difference was observed in friction between the various fractions when crushed hazelnut shells were added. For the sandpaper at a pressure of 9.33 kPa for each fraction of the nutshells, an increase in the value of this parameter within the range of 15.5–20.8% compared to the soap without the additive was recorded. For the wetted leather surface, an increase in friction was found in the case of all fractions of the nutshells added and for all ranges of analysed pressures. This may be due to the washing out of the soap fractions and the remaining undissolved nutshell particles increasing the friction resistance on the test surface. This effect is most noticeable at the highest pressure (9.33 kPa).

**Figure 8:**
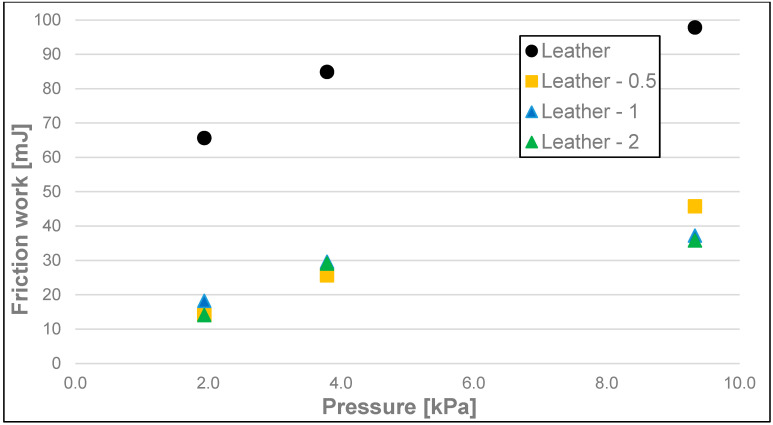
Dynamic friction work of analysed soap samples on dry synthetic leather.

**Figure 9:**
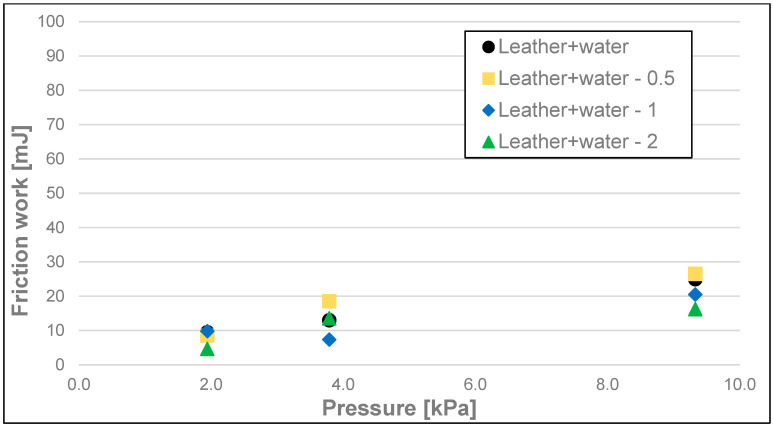
Dynamic friction work of analysed soap samples on wetted synthetic leather.

**Figure 10:**
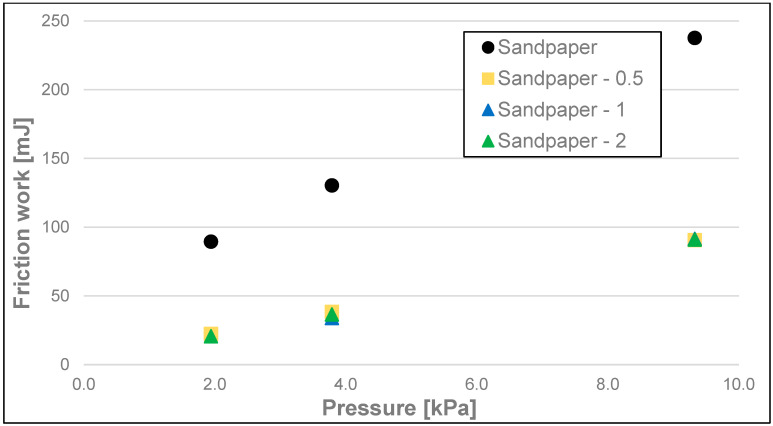
Dynamic friction work of analysed soap samples on sandpaper.

The differences between the measurements of dynamic friction work (friction work) (Figure 8, Figure 9 and Figure 10 and Table 3) of the soaps followed a similar pattern to that of dynamic friction alone, and they were most significant for the highest load, i.e., 9.33 kPa, for every tested surface. The dynamic friction work, for this highest load applied on the sample, was the smallest for the soap tested on the surface of water-moistened leather and equalled 24.96 mJ, average for the soap tested on the dry leather (99.00 mJ), and the highest for the soap tested on sandpaper (241 mJ). Significantly lower values were obtained for the medium load, and they were, respectively, 13.01 mJ for the water-moistened leather, 84.89 mJ for the dry leather, and 134 mJ for the sandpaper. At the lowest load, the work values were 9.54 mJ for the water-moistened leather, 63.64 mJ for the dry leather, and 89.92 mJ in the case of sandpaper.

**Figure 11:**
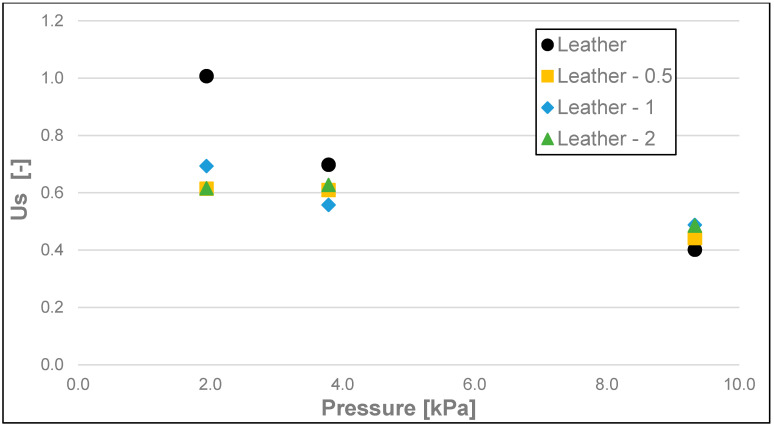
Us static friction coefficient of analysed soap samples on dry synthetic leather.

**Figure 12:**
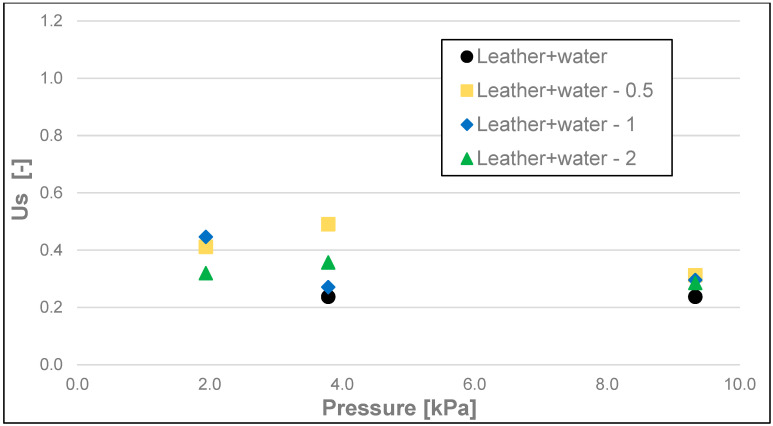
Us static friction coefficient of analysed soap samples on wetted synthetic leather.

**Figure 13:**
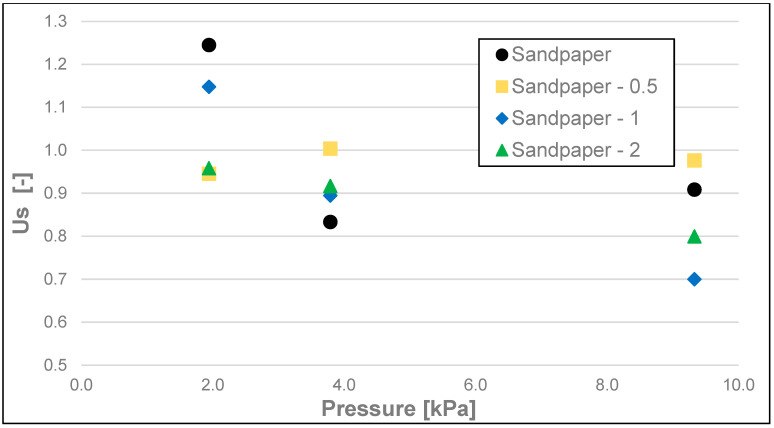
Us static friction coefficient of analysed soap samples on sandpaper.

The static friction coefficient (Us) (Figure 11, Figure 12 and Figure 13), like the static friction itself, as well as dynamic friction, was the lowest for the soap tested on water-moistened leather. It was 0.023 for the 200 g and 500 g loads and 0.040 for the 100 g load (Table 4). Significantly higher values of this coefficient were obtained for the soaps tested on dry leather and sandpaper. The static friction coefficient values for the soap tested on dry leather were 0.039, 0.073, and 0.096 for 500 g, 200 g, and 100 g loads, respectively. In contrast, the values of static friction coefficient for the soap tested on sandpaper were the highest, and they equalled 0.086, 0.080, and 0.120, respectively, for decreasing loads of 500 g, 200 g, and 100 g.

**Figure 14:**
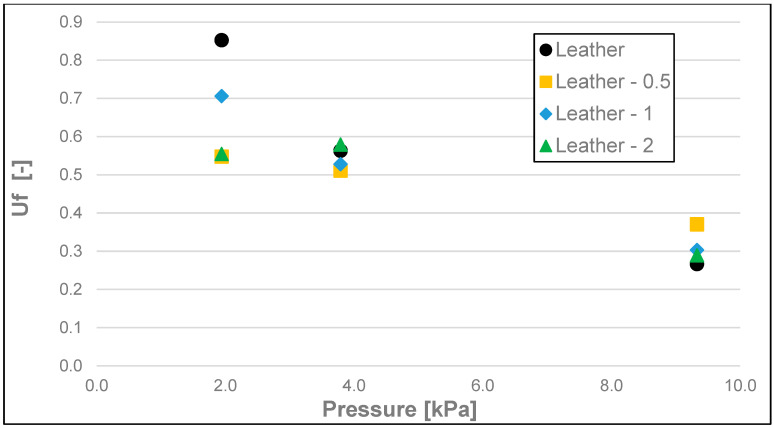
Uf dynamic friction coefficient of analysed soap samples on dry synthetic leather.

**Figure 15:**
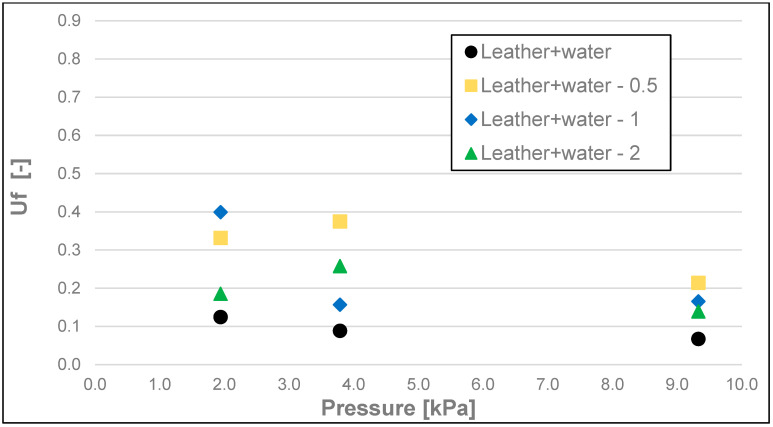
Uf dynamic friction coefficient of analysed soap samples on synthetic wetted leather.

**Figure 16:**
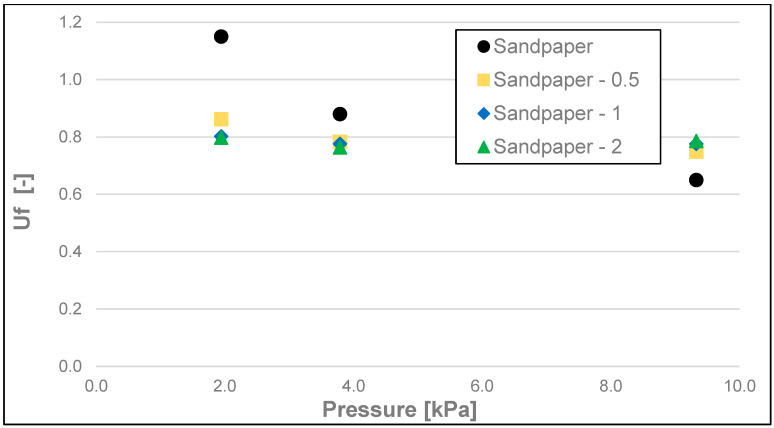
Uf dynamic friction coefficient of analysed soap samples on sandpaper.

The dynamic friction coefficient (Figure 14, Figure 15 and Figure 16 and Table 5) of the soap tested on various surfaces increased with the increasing load. As in all the examples above, the lowest values of the dynamic friction coefficient were obtained for the soap tested on water-moistened leather. They were within the range of 0.006 to 0.012. Significantly higher values of this coefficient were obtained for the soap tested on dry leather, and these were within the range of 0.039 to 0.096, while the highest values, ranging from 0.080 to 0.120, were recorded for the sandpaper surface.

**Figure 17:**
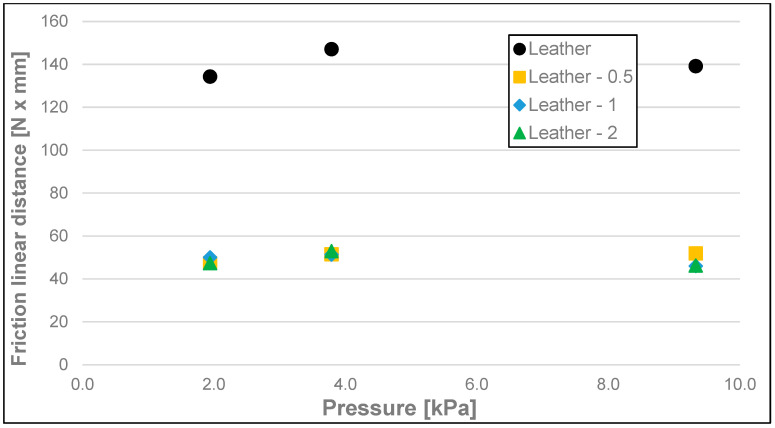
Linear dynamic friction distance of analysed soap samples on dry synthetic leather.

**Figure 18:**
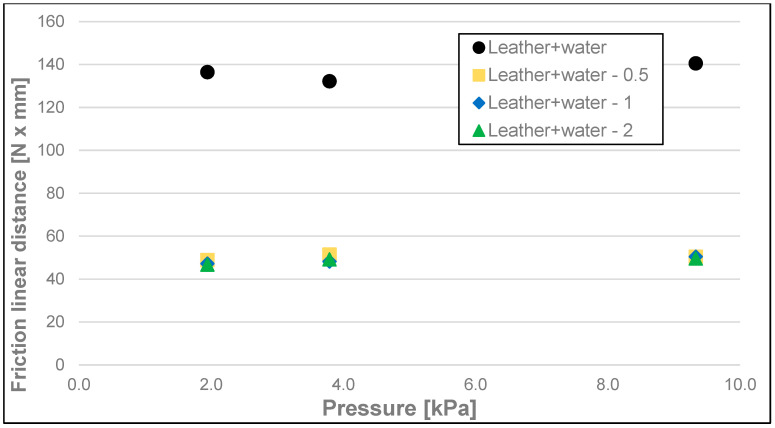
Linear dynamic friction distance of analysed soap samples on wetted synthetic leather.

**Figure 19:**
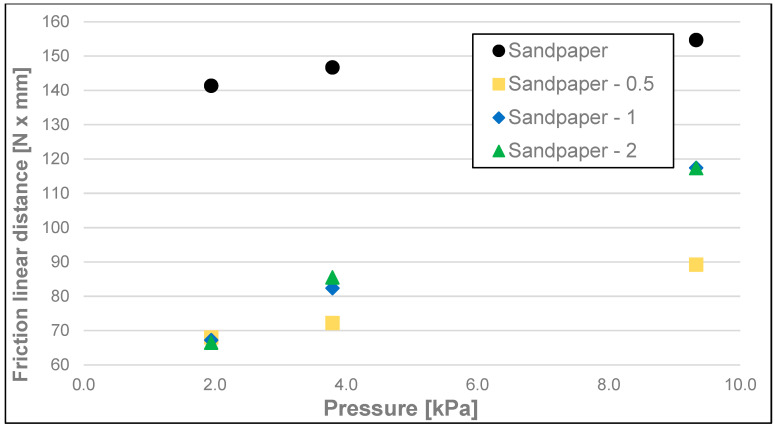
Linear dynamic friction distance of analysed soap samples on sandpaper (320).

When considering the results of linear dynamic friction distance (Figure 17, Figure 18 and Figure 19 and Table 6), in most cases, no significant differences were observed between the values of this parameter for different surfaces with different loads (500–100 g). The values of this parameter ranged from 133.2 to 154.7 N × mm.

## 4. Discussion

The study showed that the parameters of static friction, dynamic friction, and friction coefficients depend on the type of surface, i.e., dry or moistened artificial leather or sandpaper, which is consistent with our previous research, where soap without additives was tested on the same surfaces [18]. Like in other research works, the coefficient of static friction varied and was smaller when the test material was in contact with a friction pad of smaller granularity [25]. In the current study, the highest coefficient of static friction was observed for the soap on sandpaper, a smaller coefficient for the sample on dry artificial leather, while the smallest coefficient was recorded in the case of wetted artificial leather. In the study by other authors [26], it was explained that the static friction coefficient depends on the properties of the contacting surfaces, such as their microporosity, roughness, or mechanical properties of the material. Kogut and Etsion [27] proved that the external force and contact area influence static friction. In our previous study, as in the present one, the friction of the soap on water-wetted leather was the lowest, which was probably related to the reduced degree of adhesion resulting from leather moistening. Kogut and Etsion [27] indicated in their study that one of the most important parameters affecting the static friction coefficient is the degree of adhesion between bodies. Simič et al. [28] emphasised that friction depends on the level of hydration of the surface; the higher the hydration, the lower the friction. Similarly, Dong et al. [29] observed that the higher the sliding velocity, the lower the dynamic frictional force. In our previous and current studies, the highest friction was noted for the sandpaper, which would be expected given that sandpaper is often used to prevent slippage [30,31].

## 5. Conclusions

Adding ground hazelnut shells to the soaps caused a decrease in static and dynamic friction in the case of dry leather and with the lowest applied pressure (1.94 kPa) compared to the base soap, i.e., without any additives. A similar relationship can be observed when friction was applied on the surface of 320-grit sandpaper. For the applied pressure of 3.79 kPa, the additive had no statistically significant effect on static and dynamic friction in the case of the surfaces consisting of dry leather and sandpaper.

At the highest pressure (9.33 kPa), static and dynamic friction values were increased on dry leather and 320-grit sandpaper compared to the base soap (without additive). In the case of the sandpaper surface, the addition of ground hazelnut shells representing 0.5–1 mm and 1–2 mm fractions revealed a reversed relationship.

Wetting the surface of the synthetic leather resulted in a reduction in static and dynamic friction values compared to the base soap, i.e., without the additive, for each applied pressure. Wetting the surface of the leather and adding crushed hazelnut shells, regardless of the analysed fractions, at pressures of 1.94 kPa and 3.79 kPa did not cause statistically significant changes in static friction. At a pressure of 9.33 kPa, a slight increase in this value was observed. Moistening the surface of the synthetic leather caused an increase in the dynamic friction value relative to the base soap, i.e., without additive, for each applied pressure.

In the case of the dry leather surface and 320-grit sandpaper, the work of dynamic friction decreased after adding ground hazelnut shells regardless of the fraction compared to the base soap. For the wetted leather surface such a decrease was observed compared to the base soap without the additives only in the case of 1.94 kPa pressure and 1–2 mm fraction, 3.79 kPa pressure and 1–2 mm fraction, and 9.33 kPa pressure and 1–2 mm fraction.

This paper proves that it is possible to use crushed nutshells unconventionally as one of the additives in soap production. The study of the friction qualities of soaps containing ground hazelnut shells is only the beginning; more analyses of chemical properties and other parameters are designated and will be pursued in future studies. Furthermore, due to the unique methodology, various by-products are proposed to be tested as soap ingredients. This approach fits in well with responsible waste management and sustainability efforts. In general, the proposed way of using this waste group also takes into account the reduction of energy consumption and CO_2_ emissions by reducing the existing use of shells in the combustion process as an energy source.

## Figures and Tables

**Table 1:** Statistical analysis of the initial friction (stiction) of the samples as a function of surface and load applied on the sample and the addition of particular fractions of crushed hazelnut shells.

Surface	Pressure [kPa]	Size Range of Nutshell Particles Added to the Soap [mm]	Min. Stiction [N]	Max. Stiction [N]	Mean Stiction [N]	Stiction S.D.	Homogeneous Group *
Leather	1.94	-	0.93	1.21	1.04	0.10	c, d, e, f
		<0–0.5	0.58	0.68	0.63	0.05	a, b, c
		<0.5–1	0.60	0.84	0.71	0.12	a, b, c, d
		<1–2	0.59	0.68	0.63	0.04	a, b, c
	3.79	-	1.02	1.80	1.40	0.28	e, f, g, h, i
		<0–0.5	1.08	1.36	1.23	0.11	e, f, g, h
		<0.5–1	0.82	1.32	1.12	0.24	d, e, f, g
		<1–2	1.16	1.38	1.26	0.10	e, f, g, h
	9.33	-	1.66	2.40	1.99	0.32	j, k, l
		<0–0.5	1.74	2.68	2.19	0.34	k, l
		<0.5–1	2.07	2.81	2.42	0.27	l
		<1–2	2.17	2.61	2.40	0.17	l
Leather + Water	1.94	-	0.39	0.48	0.43	0.04	a
		<0–0.5	0.36	0.50	0.42	0.07	a
		<0.5–1	0.38	0.59	0.46	0.09	a
		<1–2	0.29	0.40	0.33	0.04	a
	3.79	-	0.41	0.55	0.48	0.07	a
		<0–0.5	0.81	1.15	0.99	0.12	b, c, d, e
		<0.5–1	0.43	0.61	0.54	0.07	a, b
		<1–2	0.59	0.95	0.72	0.14	a, b, c, d
	9.33	-	0.97	1.37	1.17	0.15	d, e, f, g
		<0–0.5	1.44	1.70	1.55	0.11	g, h, i, j
		<0.5–1	1.34	1.55	1.46	0.09	f, g, h, i
		<1–2	1.20	1.58	1.41	0.14	e, f, g, h, i
Sandpaper (320)	1.94	-	1.12	1.28	1.28	0.13	e, f, g, h
		<0–0.5	0.74	1.03	0.97	0.06	b, c, d, e
		<0.5–1	0.77	0.88	1.18	0.15	d, e, f, g
		<1–2	0.70	0.90	0.99	0.15	b, c, d, e
	3.79	-	1.60	2.03	1.67	0.31	h, i, j
		<0–0.5	1.47	1.73	2.02	0.18	j, k, l
		<0.5–1	1.48	1.69	1.80	0.09	i, j, k
		<1–2	1.47	1.65	1.84	0.18	i, j, k
	9.33	-	2.99	3.42	4.50	0.56	o
		<0–0.5	3.50	4.06	4.83	0.32	o
		<0.5–1	3.68	3.98	3.47	0.18	m
		<1–2	3.79	4.15	3.96	0.22	n

* a–o—the same letters indicate belonging to one homogeneous group (*p* = 0.05).

**Table 2:** Statistical analysis of dynamic friction (friction) of the samples as a function of surface and load applied on the sample and the addition of particular fractions of crushed hazelnut shells.

Surface	Pressure [kPa]	Size Range of Nutshell Particles Added to the Soap [mm]	Min. Friction [N]	Max. Friction [N]	Mean Friction [N]	Friction S.D.	Homogeneous Group *
Leather	1.94	-	0.72	1.08	0.88	0.16	f, g, h, i, j
		<0–0.5	0.53	0.63	0.56	0.04	c, d, e, f
		<0.5–1	0.55	0.92	0.73	0.14	d, e, f, g
		<1–2	0.54	0.63	0.57	0.04	c, d, e, f
	3.79	-	0.92	1.27	1.13	0.13	i, j, k, l
		<0–0.5	0.96	1.18	1.03	0.10	g, h, i, j, k
		<0.5–1	0.86	1.31	1.06	0.23	h, i, j, k
		<1–2	1.05	1.26	1.16	0.09	j, k, l
	9.33	-	1.04	1.58	1.32	0.20	k, l, m, n
		<0–0.5	1.58	2.13	1.83	0.24	p
		<0.5–1	1.39	1.56	1.50	0.07	m, n, o
		<1–2	1.35	1.53	1.43	0.07	l, m, n
Leather + Water	1.94	-	0.12	0.14	0.13	0.01	a
		<0–0.5	0.29	0.38	0.34	0.05	a, b, c
		<0.5–1	0.36	0.54	0.41	0.08	a, b, c, d
		<1–2	0.18	0.22	0.19	0.02	a, b
	3.79	-	0.16	0.20	0.18	0.02	a
		<0–0.5	0.62	0.91	0.75	0.11	e, f, g, h
		<0.5–1	0.20	0.41	0.32	0.08	a, b, c
		<1–2	0.44	0.64	0.52	0.08	b, c, d, e
	9.33	-	0.31	0.36	0.33	0.03	a, b, c
		<0–0.5	0.83	1.36	1.06	0.19	h, i, j, k
		<0.5–1	0.47	1.35	0.82	0.36	e, f, g, h, i
		<1–2	0.56	1.00	0.69	0.18	d, e, f
Sandpaper (320)	1.94	-	1.12	1.28	1.18	0.06	j, k, l, m
		<0–0.5	0.74	1.03	0.89	0.12	f, g, h, i, j
		<0.5–1	0.77	0.88	0.83	0.05	e, f, g, h, i
		<1–2	0.70	0.90	0.82	0.08	e, f, g, h, i
	3.79	-	1.60	2.03	1.77	0.16	o, p
		<0–0.5	1.47	1.73	1.58	0.10	n, o, p
		<0.5–1	1.48	1.69	1.56	0.11	n, o, p
		<1–2	1.47	1.65	1.53	0.07	n, o, p
	9.33	-	2.99	3.42	3.22	0.17	q
		<0–0.5	3.50	4.06	3.71	0.21	r
		<0.5–1	3.68	3.98	3.84	0.13	r
		<1–2	3.79	4.15	3.89	0.14	r

* a–r—the same letters indicate belonging to one homogeneous group (*p* = 0.05).

**Table 3:** Statistical analysis of dynamic friction work of the samples as a function of surface and load applied on the sample and the addition of particular fractions of crushed hazelnut shells.

Surface	Pressure [kPa]	Size Range of Nutshell Particles Added to the Soap [mm]	Min. Friction Work [N]	Max. Friction Work [N]	Mean Friction Work [N]	Friction Work S.D.	Homogeneous Group *
Leather	1.94	-	53.72	80.75	65.64	12.37	j
		<0–0.5	13.40	15.78	14.19	0.96	a, b, c, d
		<0.5–1	13.69	23.23	18.24	3.64	a, b, c, d, e
		<1–2	12.97	15.78	14.05	1.10	a, b, c, d
	3.79	-	69.21	95.02	84.89	9.87	k
		<0–0.5	23.95	29.59	25.68	2.39	d, e, f, g, h
		<0.5–1	22.72	40.77	29.63	7.47	e, f, g, h
		<1–2	26.26	31.57	29.10	2.37	e, f, g, h
	9.33	-	82.03	108.70	97.81	10.90	k
		<0–0.5	37.08	56.24	45.76	7.80	i
		<0.5–1	30.72	40.37	37.21	3.83	h, i
		<1–2	33.74	38.21	35.80	1.74	g, h, i
Leather + Water	1.94	-	9.24	9.92	9.52	0.30	a, b, c
		<0–0.5	7.00	9.61	8.51	1.21	a, b, c
		<0.5–1	9.09	11.37	9.83	0.90	a, b, c
		<1–2	4.08	5.47	4.67	0.53	a
	3.79	-	11.86	15.19	13.01	1.32	a, b, c, d
		<0–0.5	15.42	21.32	18.53	2.13	a, b, c, d, e
		<0.5–1	4.31	10.14	7.39	2.67	a, b
		<1–2	11.02	18.84	13.55	3.17	a, b, c, d
	9.33	-	22.92	27.03	24.96	1.93	d, e, f, g, h
		<0–0.5	20.68	33.94	26.53	4.82	d, e, f, g, h
		<0.5–1	11.82	33.84	20.52	9.07	b, c, d, e, f
		<1–2	11.53	25.07	16.22	5.48	a, b, c, d, e
Sandpaper (320)	1.94	-	79.41	98.46	89.52	7.73	k
		<0–0.5	19.56	25.73	22.40	2.78	c, d, e, f, g
		<0.5–1	19.18	21.89	20.66	1.24	b, c, d, e, f
		<1–2	18.48	22.39	20.73	1.52	b, c, d, e, f
	3.79	-	117.60	149.36	130.44	12.78	l
		<0–0.5	31.86	43.36	38.38	4.20	h, i
		<0.5–1	27.87	42.29	33.97	7.24	f, g, h, i
		<1–2	29.97	40.17	36.57	3.89	g, h, i
	9.33	-	226.81	248.20	237.71	8.55	m
		<0–0.5	83.73	101.44	90.67	7.03	k
		<0.5–1	80.84	99.57	91.52	8.15	k
		<1–2	78.60	103.68	91.05	10.74	k

* a–m—the same letters indicate belonging to one homogeneous group (*p* = 0.05).

**Table 4:** Statistical analysis of the Us coefficient of static friction of the samples that depends on the surface and load applied on the sample and the addition of particular fractions of crushed hazelnut shells.

Surface	Pressure [kPa]	Size Range of Nutshell Particles Added to the Soap [mm]	Min. Us	Max. Us	Mean Us	Us S.D.	Homogeneous Group *
Leather	1.94	-	0.90	1.17	1.01	0.10	l, m
		<0–0.5	0.57	0.66	0.61	0.04	f, g, h
		<0.5–1	0.58	0.82	0.69	0.11	g, h, i
		<1–2	0.57	0.66	0.62	0.04	f, g, h
	3.79	-	0.51	0.90	0.70	0.14	g, h, i
		<0–0.5	0.54	0.68	0.61	0.05	e, f, g, h
		<0.5–1	0.41	0.66	0.56	0.12	d, e, f, g
		<1–2	0.57	0.69	0.63	0.05	f, g, h
	9.33	-	0.34	0.48	0.40	0.06	a, b, c, d
		<0–0.5	0.35	0.54	0.44	0.07	b, c, d, e, f
		<0.5–1	0.42	0.57	0.49	0.05	c, d, e, f
		<1–2	0.44	0.53	0.49	0.03	c, d, e, f
Leather + Water	1.94	-	0.37	0.46	0.42	0.04	a, b, c, d, e
		<0–0.5	0.35	0.49	0.41	0.07	a, b, c, d
		<0.5–1	0.37	0.58	0.45	0.09	b, c, d, e, f
		<1–2	0.28	0.39	0.32	0.04	a, b, c
	3.79	-	0.20	0.27	0.24	0.03	a
		<0–0.5	0.41	0.57	0.49	0.06	c, d, e, f
		<0.5–1	0.22	0.30	0.27	0.04	a, b
		<1–2	0.30	0.47	0.36	0.07	a, b, c
	9.33	-	0.20	0.28	0.24	0.03	a
		<0–0.5	0.29	0.34	0.31	0.02	a, b, c
		<0.5–1	0.27	0.31	0.30	0.02	a, b, c
		<1–2	0.24	0.32	0.29	0.03	a, b
Sandpaper (320)	1.94	-	1.09	1.37	1.24	0.12	n
		<0–0.5	0.85	0.98	0.95	0.05	k, l
		<0.5–1	0.92	1.34	1.15	0.15	m, n
		<1–2	0.81	1.16	0.96	0.15	k, l, m
	3.79	-	0.64	1.02	0.83	0.16	i, j, k, l
		<0–0.5	0.91	1.11	1.00	0.09	l, m
		<0.5–1	0.86	0.95	0.89	0.05	j, k, l
		<1–2	0.83	1.04	0.92	0.09	k, l
	9.33	-	0.77	1.01	0.91	0.11	k, l
		<0–0.5	0.87	1.03	0.98	0.07	k, l, m
		<0.5–1	0.65	0.74	0.70	0.04	g, h, i, j,
		<1–2	0.75	0.84	0.80	0.05	h, i, j, k

* a–n—the same letters indicate belonging to one homogeneous group (*p* = 0.05).

**Table 5:** Statistical analysis of the dynamic coefficient of friction Uf of the samples as a function of surface and load applied on the sample and the addition of particular fractions of crushed hazelnut shells.

Surface	Pressure [kPa]	Size range of Nutshell Particles Added to the Soap [mm]	Min. Uf	Max. Uf	Mean Uf	Uf S.D.	Homogeneous Group *
Leather	1.94	-	0.70	1.05	0.85	0.16	k, l
		<0–0.5	0.51	0.61	0.55	0.04	g, h
		<0.5–1	0.53	0.89	0.71	0.14	i, j, k
		<1–2	0.52	0.61	0.55	0.04	h
	3.79	-	0.46	0.63	0.56	0.07	h, i
		<0–0.5	0.48	0.59	0.51	0.05	f, g, h
		<0.5–1	0.43	0.65	0.53	0.11	g, h
		<1–2	0.52	0.63	0.58	0.05	h, i
	9.33	-	0.21	0.32	0.27	0.04	b, c, d, e
		<0–0.5	0.32	0.43	0.37	0.05	e, f
		<0.5–1	0.28	0.32	0.30	0.01	c, d, e
		<1–2	0.27	0.31	0.29	0.01	c, d, e
Leather + Water	1.94	-	0.12	0.13	0.12	0.01	a, b
		<0–0.5	0.28	0.37	0.33	0.04	d, e
		<0.5–1	0.35	0.53	0.40	0.07	e, f, g
		<1–2	0.17	0.21	0.19	0.02	a, b, c, d
	3.79	-	0.08	0.10	0.09	0.01	a
		<0–0.5	0.31	0.45	0.37	0.05	e, f
		<0.5–1	0.10	0.20	0.16	0.04	a, b, c
		<1–2	0.22	0.32	0.26	0.04	b, c, d, e
	9.33	-	0.06	0.07	0.07	0.01	a
		<0–0.5	0.17	0.27	0.21	0.04	a, b, c, d
		<0.5–1	0.10	0.27	0.17	0.07	a, b, c
		<1–2	0.11	0.20	0.14	0.04	a, b
Sandpaper (320)	1.94	-	1.09	1.24	1.15	0.06	m
		<0–0.5	0.72	1.00	0.86	0.12	l
		<0.5–1	0.74	0.85	0.80	0.05	k, l
		<1–2	0.68	0.87	0.80	0.07	j, k, l
	3.79	-	0.79	1.01	0.88	0.08	l
		<0–0.5	0.73	0.86	0.78	0.05	j, k, l
		<0.5–1	0.73	0.84	0.78	0.05	j, k, l
		<1–2	0.73	0.82	0.76	0.03	j, k, l
	9.33	-	0.60	0.69	0.65	0.03	h, i, j
		<0–0.5	0.71	0.82	0.75	0.04	j, k, l
		<0.5–1	0.74	0.80	0.78	0.03	j, k, l
		<1–2	0.77	0.84	0.79	0.03	j, k, l

* a–m—the same letters indicate samples belonging to one homogeneous group (*p* = 0.05).

**Table 6:** Statistical analysis of the linear dynamic friction distance of the samples as a function of surface and load applied on the sample and the addition of particular fractions of crushed hazelnut shells.

Surface	Pressure [kPa]	Size Range of Nutshell Particles Added to the Soap [mm]	Min. Linear Distance of Friction [N × mm]	Max. Linear Distance of Friction [N × mm]	Mean Linear Distance of Friction [N × mm]	Linear Distance of Friction S.D.	Homogeneous Group *
Leather	1.94	-	130.18	138.58	134.35	3.45	g, h
		<0–0.5	46.59	48.13	47.33	0.56	a
		<0.5–1	47.27	52.41	50.10	2.35	a
		<1–2	46.96	48.01	47.37	0.41	a
	3.79	-	139.57	152.82	147.09	6.87	h, i
		<0–0.5	46.50	56.22	51.53	3.81	a
		<0.5–1	49.17	53.50	51.33	1.91	a
		<1–2	49.12	56.62	52.98	3.56	a, b
	9.33	-	136.03	142.55	139.17	2.78	g, h
		<0–0.5	48.34	56.45	51.89	3.13	a
		<0.5–1	44.60	47.71	46.08	1.41	a
		<1–2	43.79	48.59	46.28	1.93	a
Leather + Water	1.94	-	131.05	140.17	136.48	3.85	g, h
		<0–0.5	46.23	50.69	48.91	1.93	a
		<0.5–1	45.88	48.78	47.21	1.14	a
		<1–2	44.75	48.97	46.75	1.87	a
	3.79	-	129.18	135.56	132.20	2.98	g
		<0–0.5	46.03	55.70	51.45	4.61	a
		<0.5–1	44.36	51.34	48.18	3.43	a
		<1–2	45.24	53.20	49.16	3.43	a
	9.33	-	133.13	143.49	140.55	4.23	g, h
		<0–0.5	46.40	52.97	49.59	2.69	a
		<0.5–1	46.22	62.63	50.45	6.84	a
		<1–2	46.40	52.97	49.59	2.69	a
Sandpaper (320)	1.94	-	131.66	148.77	141.35	6.43	g, h, i
		<0–0.5	62.03	77.82	68.03	6.34	c
		<0.5–1	61.00	70.48	67.24	4.05	c
		<1–2	55.30	73.51	66.49	8.03	b, c
	3.79	-	135.71	159.65	146.71	10.94	h, i
		<0–0.5	65.54	80.89	72.23	6.66	c, d
		<0.5–1	79.01	92.82	82.37	5.90	d, e
		<1–2	73.05	95.05	85.51	9.44	d, e
	9.33	-	146.39	164.03	154.69	6.90	i
		<0–0.5	79.69	104.57	89.22	9.53	e
		<0.5–1	103.74	135.75	117.43	12.82	f
		<1–2	99.44	130.48	117.34	12.80	f

* a–i—the same letters indicate samples belonging to one homogeneous group (*p* = 0.05).

## Data Availability

The original contributions presented in the study are included in the article, further inquiries can be directed to the corresponding authors.
